# Microbial Characteristics of Common Tongue Coatings in Patients with Precancerous Lesions of the Upper Gastrointestinal Tract

**DOI:** 10.1155/2022/7598427

**Published:** 2022-04-18

**Authors:** Xiaoyu Kang, Bin Lu, Pan Xiao, Zhaolai Hua, Rui Shen, Jianping Wu, Juan Wu, Zhenfeng Wu, Chun Cheng, Junfeng Zhang

**Affiliations:** ^1^School of Medicine & Holistic Integrative Medicine, Nanjing University of Chinese Medicine, Nanjing 210000, China; ^2^Department of Oncology, Yangzhong People's Hospital, Yangzhong, Jiangsu 212200, China; ^3^Laboratory Animal Centre, Nanjing University of Chinese Medicine, Nanjing 210000, China; ^4^Department of Surgical Oncology, Jiangsu Province Hospital of Chinese Medicine, Affiliated Hospital of Nanjing University of Chinese Me dicine, Nanjing 210029, China

## Abstract

The tongue coating (TC) microbiota, a crucial component of the tongue coating, illustrates a huge microbial percentage of the body that mostly includes actinobacteria, bacteroides, firmicutes, and fusobacteria. The TC microbiota is closely related to the development of upper gastrointestinal malignancies, such as oral, gastric, and esophageal cancer. Nonetheless, the microbiological characteristics of common TCs in individuals with precancerous lesions of the upper gastrointestinal tract are still unclear. Herein, we designed a case-control study, recruiting 153 PLUGT patients with four different types of TCs, including 47 white-thin, 19 white-thick, 47 yellow-thin, and 40 yellow-thick, as well as 47 volunteers as controls. To analyze microbial characteristics, 16S rRNA microbiome approaches were used. An enzyme-linked immunosorbent assay (ELISA) was employed to assess serum IL-17A and total bile acid (TBA). According to the obtained results, *Leptotrichia* was found to be a promising biomarker for thin as well as thick yellow coatings. In comparison to the control TC microbiota, 39 different genera developed commensal networks in common TCs. *Lachnoanaerobaculum* and *pseudonocardia* were the most striking core bacteria. *Lachnoanaerobaculum* positively correlated with *Leptotrichia* in W-thin and Y-thick coatings, with *actinomyces* and *methylobacterium* in Y-thin coatings, with *Campylobacter* in Y-thick coatings, and with *Bradyrhizobium* in W-thick and Y-thick coatings. Serum IL-17A levels were greater in cases with W-thin coating than in controls, and serum IL-17A was positively linked with *Parvimonas* in patients with W-thick or Y-thin coating. In Y-thin coating, the oral dominating bacteria *Streptococcus* was negatively linked with serum TBA. Taken together, the promoted bacteria were found to be synergistically proliferative in the TCs of PLUGT patients. The diverse TCs had distinct bacterial commensal networks, whereas the common TCs were linked by specific bacteria to serum IL-17A and TBA.

## 1. Introduction

The upper gastrointestinal tract (GIT) includes the oral cavity, pharynx, esophagus, stomach, and duodenum. Gastric cancer (GC) and esophageal cancer (EC) are two of the most often seen cancers in clinical practice. Globally, GC is the third major reason for deaths related to cancer and the fifth most abundant sort of cancer, whereas EC ranks sixth in mortality and seventh in morbidity [1]. GC has the third greatest incidence and mortality rate of all malignant tumors in China, while EC has the seventh greatest incidence and the rate of mortality [2]. Numerous studies have demonstrated that early recognition and diagnosis of upper GIT cancer are critical strategies for lowering mortality and incidence rates and considerably increasing survival rates up to 5 years [3–5]. Precancerous lesions of the upper gastrointestinal tract (PLUGT) are a crucial stage in the transformation of the normal upper gastrointestinal mucosa into upper gastrointestinal cancer. Atrophy of the gastric glands associated with abnormal hyperplasia and/or intestinal metaplasia of the gastric mucosal epithelium is regarded as a precancerous lesion of gastric cancer (PLGC) [6, 7]. PLGC is the intermediate link in the transformation from gastritis to gastric cancer, and it is essential for reducing GC risk to timely and effective block or reverse the PLGC [8, 9]. Esophageal squamous cell carcinoma (ESCC) accounts for more than 85% of EC globally [10, 11]. ESCC precancerous and cancerous lesions are graded as follows: severe dysplasia/carcinoma, moderate dysplasia, mild dysplasia, and basal cell hyperplasia in situ [12]. Currently, the diagnosis of GC and EC initially depends on endoscopy and pathological biopsy, which has the characteristics of the invasive injury and high cost and leads to fear in patients; therefore, most patients with upper digestive tract cancer are clinically diagnosed at an advanced stage [13–15].

Tongue diagnosis is objective, simple, noninvasive, and rapid in traditional Chinese medicine (TCM) diagnostic methods and has a lengthy background. The holistic view of TCM believes that variable tongue coating (TC) is capable of reflecting the pathophysiological function of the spleen and stomach, which is mainly corresponding to digestive function [16–18]. Tongue diagnosis has great application value in many systemic diseases, systematically summarized in the classical book “*Bianshe Zhinan*” (referred to *Guide to Tongue Diagnosis*). In clinical TCM practices, TC is the key content of tongue diagnosis, and its characteristic (including thickness and color) is an important basis for syndrome differentiation and treatment for many digestive system diseases. The four most common syndromes are exterior cold syndrome, interior cold syndrome, exterior heat syndrome, and interior heat syndrome which are most commonly associated with white-thin (W-thin), white-thick (W-thick), yellow-thin (Y-thin), and yellow-thick (Y-thick) TCs, respectively.

Modern studies believe that TC consists of microorganisms (mainly bacteria and fungi), exfoliated epithelium, blood metabolites, and saliva derived from filamentous papilla [19]. A growing body of research in recent years has revealed a relationship between the TC microbiota and several systemic illnesses, such as malignant tumors and diabetes. The occurrence and progression of systemic disorders, as well as the treatment process, can be dynamically reflected by TC type and its microbiota. The tongue coating microbiota has been established as a possible gastritis biomarker [20] and has been shown to have prognostic significance for pancreatic head cancer [21] and gastric cancer [20]. The changes in the TC microbiota may be related to inflammation and the metabolome, and the bacteria can potentially act as a noninvasive biomarker for GC diagnosis, independent of lifestyle [22]. The tongue coating microbiota changes as halitosis progresses, and assessing tongue coating microbiome biomarkers could help predict the risk of halitosis in children [23]. Ye et al. [24] revealed a genus *Bacillus* that existed exclusively on the yellow tongue coating of cases suffering from chronic erosive gastritis. Both TC type and its microbiota can dynamically reflect the occurrence and development of systemic diseases and the treatment process. Cui et al. [20] found that the prevalence of TC *Campylobacter concisus* was significantly greater in intestinal metaplasia superficial gastritis, and atrophic gastritis compared to normal controls, and that the prevalence of *C. concisus* gradually increased with the progression of gastritis, including the precancerous cascade. Furthermore, TC microbiota has the potential to serve as biomarkers for several different types of systemic diseases, including malignant tumors [18, 25]. Wu et al. [26] found that the GC risk was related to increased *Firmicutes* and decreased *Bacteroidetes*. Xu et al. [22] found that the six TC bacterial genera combinations (*Fusobacterium, Megamonas*, *Rothia, Porphyromonas*, *Peptostreptococcus*, and *Peptococcus*) had a strong diagnostic value for GC risk. These results are further proof of the outstanding ability of tongue diagnosis in many systemic diseases besides malignant tumors. However, the microbial characteristics of the common TCs are still unclear.

Bacteria promote tumor formation by producing carcinogenic metabolites and inhibiting chronic and persistent inflammation, especially in organs exposed to microorganisms for a long time [27, 28]. Numerous studies showed that bile acid promoted intestinal metaplasia and GC [29, 30]. Karstens et al. [31] explored that the IL-17A level was reduced in cases with esophageal adenocarcinomas in comparison to healthy controls. And our previous study found that GC patients demonstrated a high level of serum IL-17A than the controls, and the patients with white coating had substantially lesser serum IL-17A levels compared to those with yellow coatings [32].

Therefore, this study comprehensively analyzed the microbial characteristics and potential biomarkers of common TC (Y-thin, Y-thick, W-thin, and W-thick) in PLUGT patients and explored the relationship between the different TC bacteria and the serum IL-17A and TBA. The results are expected to provide precise methods for the rapid and noninvasive diagnosis of PLUGT and embody the scientific connotation of tongue-diagnosis-assisting TCM syndrome differentiation and treatment.

## 2. Patients and Methods

### 2.1. Study Participants

The Clinical Ethics Committee at Yangzhong People's Hospital in Jiangsu Province (No. PHYC2018039) approved this study. From April 2019 to January 2020, the unified questionnaire was utilized for collecting clinical information from the upper gastrointestinal cancer screening of residents aged 40 to 70 in Yangzhong City. Endoscopy and pathologic biopsy were used to diagnose all PLUGT patients, who were histologically confirmed to have pancreatic lesions of gastric cancer (PLGC) and/or precancerous lesions of esophageal squamous cell carcinoma (ESCC). A total of 153 PLUGT patients were included in the case group, whereas 47 white-thin coating volunteers comprised the healthy control group. At the time of enrollment, all participants provided signed informed permission. All participants were excluded on the basis of the following criteria: (1) all participants who got probiotics, antibiotics, or both within the preceding 4 weeks and were diagnosed with periodontitis or another oral disease; (2) had any malignant tumor; (3) had a history of severe systemic diseases (Alzheimer's disease, liver cirrhosis, ulcerative colitis, etc.); and (4) unwillingness to cooperate. Furthermore, participants in the control group with severe digestive diseases, and significant gastrointestinal discomforts, including stomach pain, diarrhea, and abdominal pain, were excluded.

### 2.2. Sample Collection

All contributors were required to fast during the night hours (≥8 hours) and gargle with normal saline 2 to 3 times in the morning. Two traditional Chinese physicians with a combined clinical experience of >10 years independently diagnosed the TC type, and the TC images were photographed and analyzed using the DS01-B tongue diagnostic information acquisition system (DAOSH Co., Ltd, Shanghai, China). The patients were fit into this study when two physicians' diagnoses and DS01-B analysis were consistent. A sterile one-off toothbrush was used to collect each TC sample along the median groove of the tongue dorsum and suspended it into the sterile normal saline. After centrifugation of 5 min at 3000 r/min, the precipitates were collected as TC samples. All participants' peripheral blood (3–5 mL) was drawn and centrifuged for 10 min at 3000 r/min to achieve the serum. All the specimens were kept at −80°C within 2 h.

### 2.3. DNA Extraction, Polymerase Chain Reaction (PCR) Amplification, and Sequencing

The extraction of total TC DNA was performed in accordance with the manufacturer's procedure that used QIAamp DNA Mini Kit (Qiagen, Valencia, CA, USA). The V3–V4 region of the 16S (rRNA gene) was amplified employing the forward primer 341F (5′-CCTAYGGGRBGCASCAG-3′) and the reverse primer 806R (5′-GGACTACNNGGGTATCTAAT-3′). The PCR conditions were identical to those used in our previous study [19]. Electrophoresis on a 2% agarose gel was implemented to identify the products of PCR. AxyPrep DNA Gel Extraction Kit (Axygen Biosciences, CA, USA) was used to extract PCR products, and a QuantiFluor^TM^-ST Assay Kit (Promega, WI, USA) was used to quantify them. The pooled DNA product was utilized for preparing the Illumina pair-end library in the same manner as the Illumina genomic DNA library preparation technique. Following that the library of the amplicon was sequenced paired-end on an Illumina MiSeq platform (Shanghai Biozeron Co., Ltd, Shanghai, China) employing standard guidelines. QIIME version 1.17 was employed to filter and remove low-quality sequencing reads (<200 bp), and UPARSE version 7.1 was implemented to cluster operational taxonomic units (OTUs) with 97% similarity cut-off after removal of chimeric sequences (http://drive5.com/uparse/). Taxonomic assessment was performed using the SILVA database, and each OUT was classified into the following five categories (phylum, class, order, family, and genus).

### 2.4. Laboratory Detection

Serum interleukin (IL)-17A (Human IL-17A ELISA KIT, ZC-32330) and total bile acid (TBA; Total Bile Acid ELISA Kit, MM-50350H1) levels were determined employing enzyme-linked immunosorbent assay (ELISA). All procedures were performed in metculous compliance with the manufacturer's instructions.

### 2.5. Bioinformatics and Statistical Analyses

The alpha diversity, community structure, and linear discriminant assessment of the effect size (LEfSe) were executed using the computer program Visual Genomics (Release1, Shanghai Infinity Biotechnology Co., Ltd, Shanghai, China). SPSS software version 26.0 was employed for statistical assessment. The student's *t*-test was implemented for normal distribution data, which were expressed as the mean ± standard deviation. The chi-square analysis was implemented for the grade data. Non-normal distribution data were assessed applying the Mann–Whitney *U* nonparametric assessment and represented as the median (interval value). The symbiotic networks were drawn using Spearman's correlation analysis and Cytoscape software (https://cytoscape.org/). GraphPad Prism 8 computer program was employed for drawing the point diagrams. The statistical result was two-sided, and *P* < 0.05 indicated statistically meaningful differences. Potential alterations in the microbiome at the functional level were appraised by implementing PICRUSt computer program and the Kyoto Encyclopedia of Genes and Genomes (KEGG) databank.

## 3. Results

The following section describes the core findings of the study.

### 3.1. Characteristics of the Study Population

A total of 153 PLUGT patients were categorized into four categories on the basis of the color and type of their TC (1) The W-thin group (*n* = 47) consisted of women and men (30 and 17), with an average age of 57.3 ± 7.2 years; (2) W-thick group (*n* = 19) included men and women (8 and 11), with an average age of 57.8 ± 8.4 years; (3) Y-thin (*n* = 47) included men and women (17 and 30), with an average age of 57.3 ± 7.7 years; and (4) Y-thick (*n* = 40) consisted of men and women (31 and 9), with an average age of 60.4 ± 5.8 years. The healthy controls (*n* = 47) included men and women (16 and 31), with an average age of 54.7 ± 8.8 years. In terms of age and sex, the W-thin, W-thick, and Y-thin groups have no meaningful differences with the control group (*P* > 0.05); however, the difference is significant in the case of the Y-thick group (*P* < 0.05).

### 3.2. Diversity of the Bacterial Community

Alpha diversity focuses on the richness and diversity of the microflora. Chao and ACE indices were employed to predict operational taxonomic units (OTUs) richness, whereas Shannon, Simpson, and Observed OTUs indices were implemented to appraise OTUs diversity. The Mann–Whitney *U* analysis was employed to scrutinize the discrepancy in alpha diversity between the control group and the four PLUGT patient groups (Table S1). Among the total five groups, there are meaningful discrepancies (*P* < 0.05) in Chao, Shannon, and Observed OTUs (*P* < 0.05). Compared with the control group, ACE, Chao, Shannon, and Observed OTUs of microorganisms in PLUGT patients were considerably enhanced (*P* < 0.05), whereas Simpson was considerably diminished (*P* < 0.05). In the W-thin and Y-thick groups, ACE, Chao, Shannon, and Observed OTUs enhanced substantially (*P* < 0.05), whereas Simpson reduced notably (*P* < 0.05). In the Y-thin group, Chao, Shannon, and Observed OTUs increased significantly (*P* < 0.05), whereas Simpson diminished remarkably (*P* < 0.05). The results suggested that the TC type was closely associated with the bacterial richness and diversity in PLUGT cases.

### 3.3. The Bacterial Community Structure of the Tongue Coating

**Figure 1:**
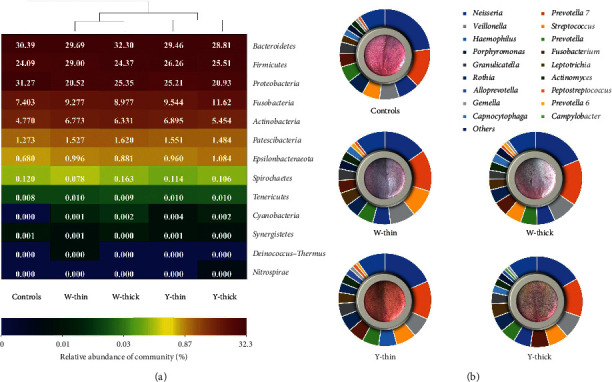
The composition and clustering analysis of TC microbiota in PLUGT patients and the controls. (a) The phylum-level heatmap. Cluster analysis is conducted based on the distance calculation using the Bray–Curtis method, and the results show that the same color TCs have similar microbial structures. (b) The genus-level doughnut chart. The size of bars represents the mean relative abundance of dominant bacterial genera. Each genus is represented by a different color. The image in the center of each doughnut chart is the typical TC.

Across all the TC samples, 13,774 OTUs belonged to 33 phyla, 75 classes, 189 orders, 268 families, and 657 genera. The predominant bacterial phyla and genera (relative abundance >1%) were similar among the five groups (Figure 1). At the phylum level, the cluster analysis found that all samples were categorized into two subgroups: the control group and the PLUGT group, and the bacteria of W-thin and W-thick got together, while the bacteria of Y-thin and Y-thick got together in the PLUGT group (Figure 1(a)). It was suggested that phyla-level bacterial composition was associated with the TC color transformation from white to yellow in PLUGT patients. Eighteen dominant bacterial genera (*Neisseria*, *Prevotella 7*, *Veillonella*, *Streptococcus*, *Haemophilus*, *Prevotella*, *Porphyromonas*, *Fusobacterium*, *Granulicatella*, *Leptotrichia*, *Rothia*, *Actinomyces*, *Alloprevotella*, *Peptostreptococcus*, *Gemella*, *Prevotella 6*, *Capnocytophaga*, and *Campylobacter*) were observed in the five kinds of TCs (Figure 1(b)).

### 3.4. Linear Discriminant Analysis (LDA) of the Microbiome of Tongue Coating

**Figure 2:**
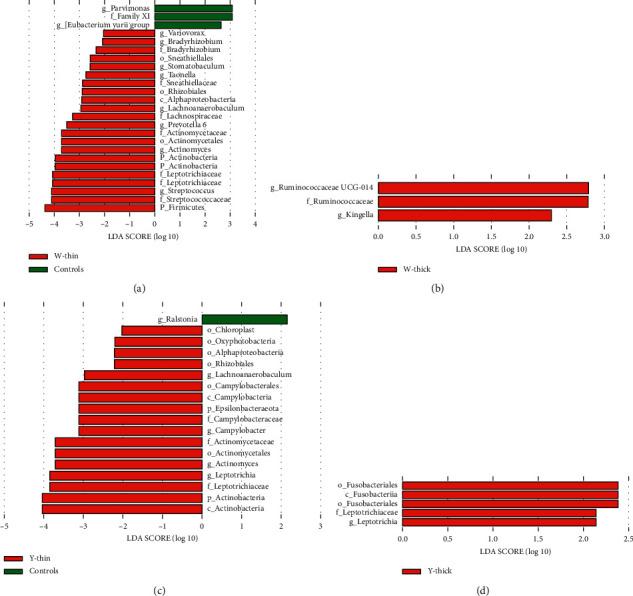
LDA assessment of TC microbiota between PLUGT patients and controls. Based on the controls, LDA analysis is conducted to screen potential biomarkers related to PLUGT with common TCs: (a) Y-thin group, (b) W-thick group, (c) Y-thin group, and (d) Y-thick group. Generally, LDA score means the significance of the microbial taxa. “*p*” refers to phylum, “*c*” refers to a class, “*o*” refers to order, “*f*” refers to family, and “*g*” refers to a genus.

Based on the microorganisms of the controls, LDA was used to identify potential biomarkers for regular TCs in PLUGT cases. There were 5, 18, 3, and 25 marker bacterial taxa in the Y-thick groups, Y-thin, W-thick, and W-thin, accordingly. At the genus level, there were 10 potential biomarkers in the W-thin group: *Variovorax*, *Bradyrhizobium*, *Stomatobaculum*, *Taonella*, *Lachnoanaerobaculum*, *Prevotella 6*, *Actinomyces*, *Streptococcus*, *Parvimonas*, and *(Eubacterium) yurii group* (Figure 2(a)), *Ruminococcaceae UCG-014,* and *Kingella* were the potential biomarkers in the W-thick group (Figure 2(b)), there were five potential biomarkers in the Y-thin group: *Lachnoanaerobaculum*, *Campylobacter*, *Actinomyces*, *Leptotrichia*, and *Ralstonia* (Figure 2(c)), and *Leptotrichia* was a potential biomarker in the Y-thick group (Figure 2(d)). Therefore, *Leptotrichia* might be a potential biomarker of Y-thin and Y-thick groups in PLUGT cases.

**Figure 3:**
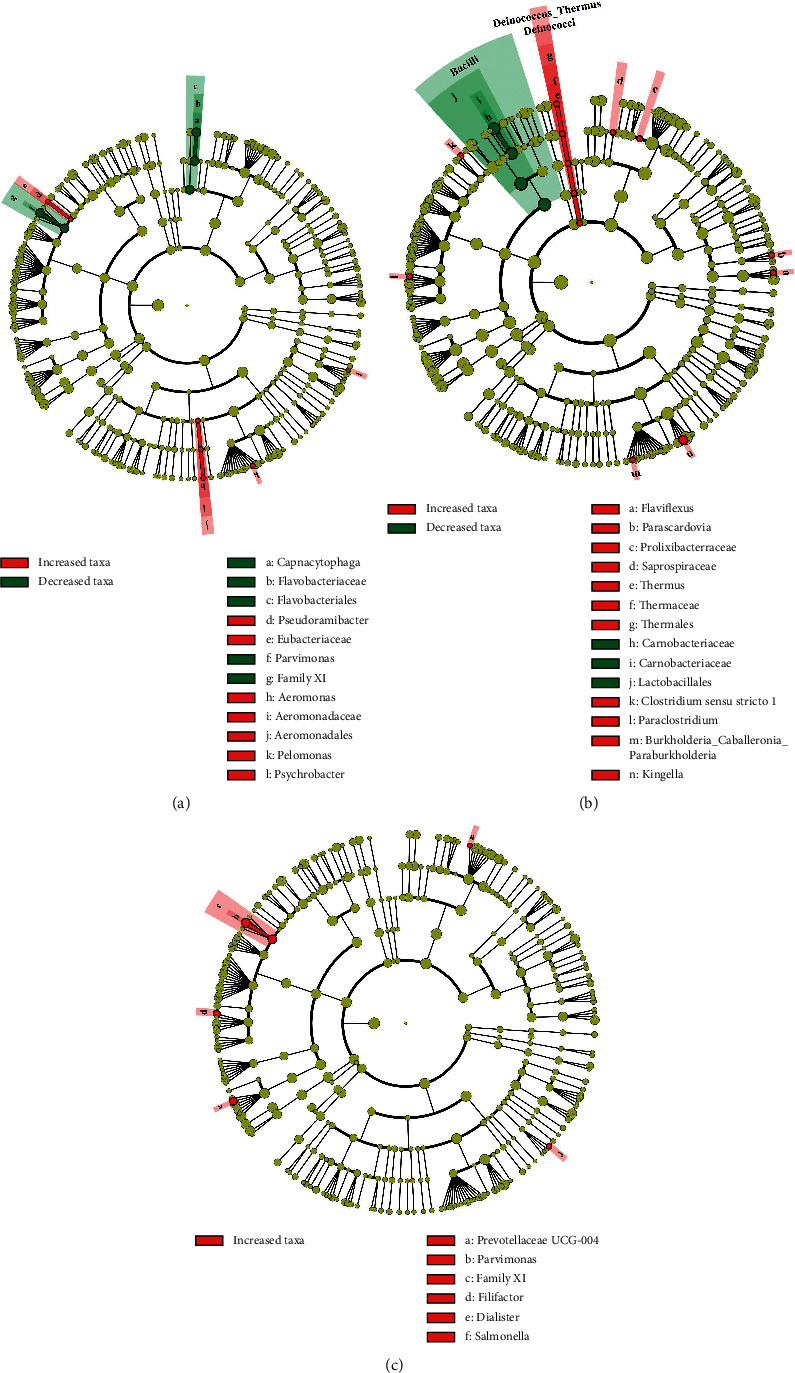
LEfSe analysis of TC microbiota in PLUGT patients. Each TC group is chosen for comparing with the other three converged groups in PLUGT patients. The LEfSe cladogram includes 5 layers of circles, which demonstrate 5 classifications (phylum, class, order, family, and genus) from the center to edge. (a) W-thin TC, (b) W-thick TC, and (c) Y-thick TC.

To further study the relationship between coating color and TC microbiota, each group of TC was chosen for comparison with the other three converged groups. The microbial compositions among the four tongue coatings were further analyzed using the LefSe. LefSe analysis analyzed microbial dysbiosis, representing substantial discrepancies in the abundance of bacterial populations between the various groups. As a result of LefSe analysis, 32 bacterial taxa were identified. Among them, the relative abundances of 24 and 8 bacterial taxa were substantially increased and decreased, respectively (Figure 3). The comparative abundances of four bacterial genera (*Psychrobacter*, *Pelomonas*, *Aeromonas*, and *Pseudoramibacter*) were remarkably increased in the W-thin group (Figure 3(a)); the relative abundances of seven bacterial genera (*Clostridium sensu stricto 1*, *Burkholderia_Caballeronia_Paraburkholderia*, *Kingella*, *Parascardovia*, *Flaviflexus*, *Paraclostridium*, and *Thermus*) were significantly increased in the W-thick group (Figure 3(b)); the relative abundances of five bacterial genera (*Parvimonas*, *Prevotellaceae UCG-004*, *Salmonella*, *Dialister*, and *Filifactor*) were significantly increased in the Y-thick group (Figure 3(c)). However, no unique genus was found in the Y-thin group, which might have a similar bacterial community structure as the Y-thick group. The aforementioned results indicated that TC microbiota was closely related to the formation of TC color.

### 3.5. The Symbiotic Relationship of the TC Microbiome

**Figure 4:**
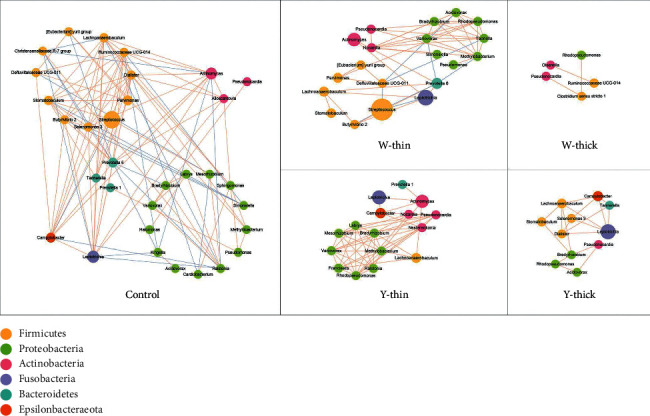
The symbiotic relationship of distinct genera from different TCs.

In a normal circumstance, mixed bacteria have complex symbiotic relationships for maintaining the normal micro ecological balance, but in the disease condition, bacterial dysbiosis results in a distinct symbiotic relationship. Therefore, the symbiotic relationship may present a novel entry point for comprehending the microecological mechanism of disease occurrence. According to the controls' white-thin coating microbiota, Mann–Whitney *U*-test was conducted to screen the distinct bacterial genera in the four pathological TCs (Table S2). Spearman's association assessment was executed on the basis of distinct genera of each group, and the meaningful associations (*P* < 0.05) were presented as symbiotic networks (Figure 4). As shown in Figure 4, each node represents a genus and the genus's average relative abundance is represented by the size of each node. Nodes of the same color represent members of the same phyla. Edges illustrate relationships with values superior to 0.5 and *P* values lower than 0.05. Generally, most correlations were significantly positive, and only in W-thin coating did the negative correlations appear. As evident from the figure, the W-thick coating manifested the least number of correlations. In the PLUGT patients, the striking *Lachnoanaerobaculum* had the most complex symbiotic relationships. It was found to correlate positively with *Leptotrichia* in W-thin and Y-thick coatings, with *Actinomyces* and *Methylobacterium* in Y-thin coating, with *Campylobacter* in Y-thick coating, and with *Bradyrhizobium* in W-thick and Y-thick coatings. *Pseudonocardia* was the other significant promoted bacterial genus in the four common TCs, it positively correlated with *Methylobacterium* in W-thin coating, and positively correlated with *Variovorax* and *Bradyrhizobium* in Y-thin coating. As shown in Figure 4, the *Prevotella* spp was found to have negative associations with *Methylobacterium and Variovorax. Leptotrichia* demonstrated negative correlations with *Streptococcus.* These results suggested that the promoted bacteria were synergistic proliferative.

Each node is related to a distinct genus. The size of each node illustrates the mean relative abundance of each genus. Nodes of the same color come from the same phylum. Relationships with values superior to 0.5 and *P* values lower than 0.05 are demonstrated as edges. The red edges mean a positive relationship and the blue edges mean a negative relationship.

### 3.6. Predictive Functional Analysis of the TC Microbiome

Potential biological functions of TC microbiota were evaluated using the KEGG database. Employing the Mann–Whitney *U-*test, four predictive functions were significantly different between the four PLUGT patients' groups in comparison to the control group (*P* < 0.05). They were betalain biosynthesis, fluorobenzoate degradation, indole alkaloid biosynthesis, and vasopressin-regulated water absorption (Figure S1). Using LDA, we found that changes in the five predictive functions of the Y-thick group also existed in the W-thin and Y-thin groups, including three reduced predictive functions (ABC transporters, phosphotransferase system, and transporters) and two enhanced predictive functions (chaperones and folding catalysts, and chromosome; Figure S2). However, no predictive function was found in the W-thick group, suggesting that a W-thick TC might be the basic pathological TC in PLUGT patients.

**Figure 5:**
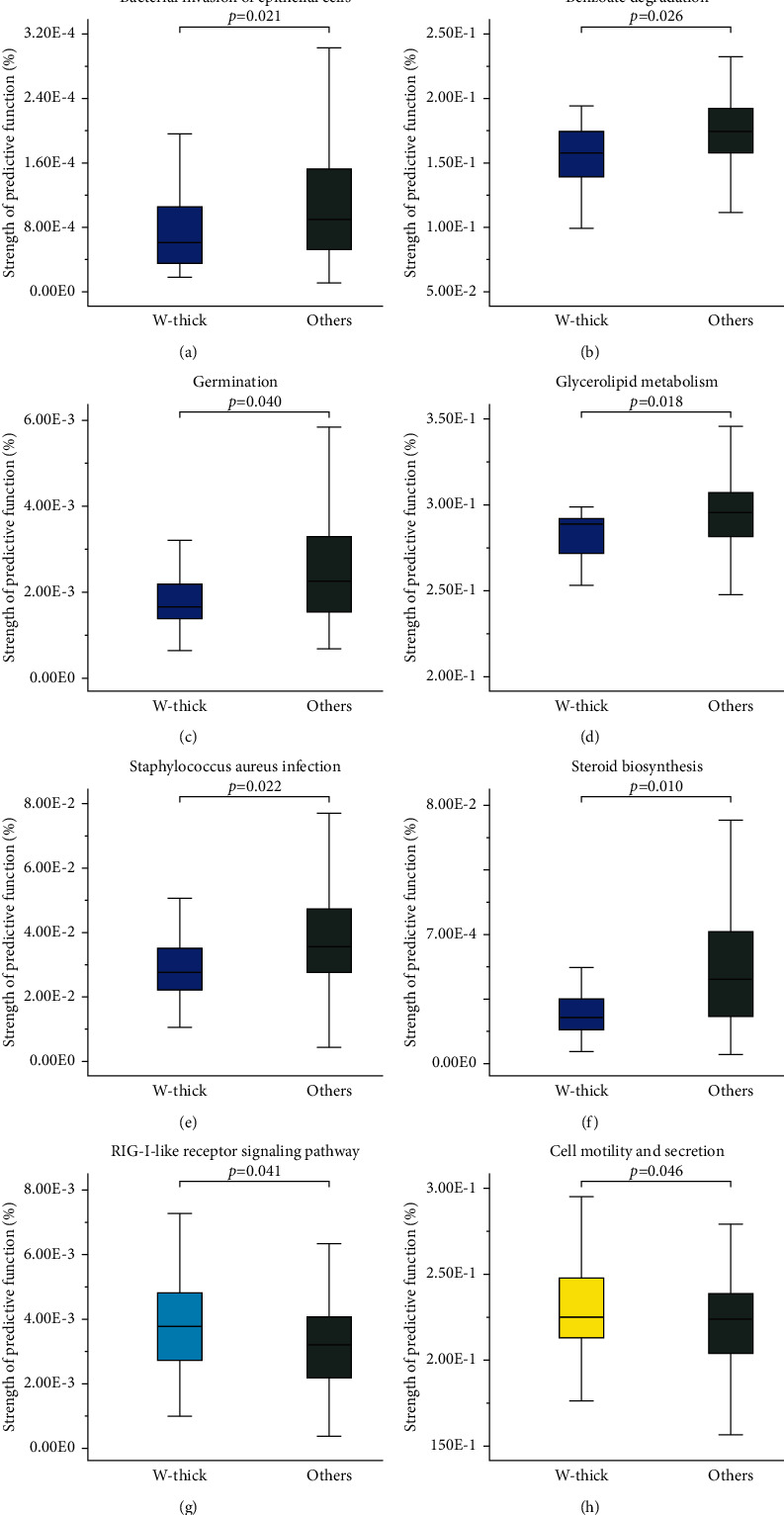
Eight predictive functions with significant differences based on TC microbiota in PLUGT patients. Each TC group is chosen for comparing with the other three converged groups in PLUGT patients. Differences between subgroups are evaluated through the Mann–Whitney *U*-test (only *P* < 0.05 are indicated).

To further explore the unique functions of the four common TCs in PLUGT patients, each TC group was chosen for comparison with the other three converged groups. The Mann–Whitney *U* assessment revealed that the eight predictive functions were significantly different (Figure 5). Among them, the W-thick group manifested six significantly enhanced functions in comparison to the other three converged groups. These included the bacterial invasion of epithelial cells, benzoate degradation, germination, glycerolipid metabolism, *Staphylococcus aureus* infection, and steroid biosynthesis (Figure 5(a)–5(f)). The RIG-I-like receptor (RLR) signaling pathway was significantly enhanced in the W-thin group (Figure 5(g)). Cell motility and secretion were significantly enhanced in the Y-thick group (Figure 5(h)).

### 3.7. Serum IL-17Aand TBA Correlated with Distinct TC Bacteria

**Figure 6:**
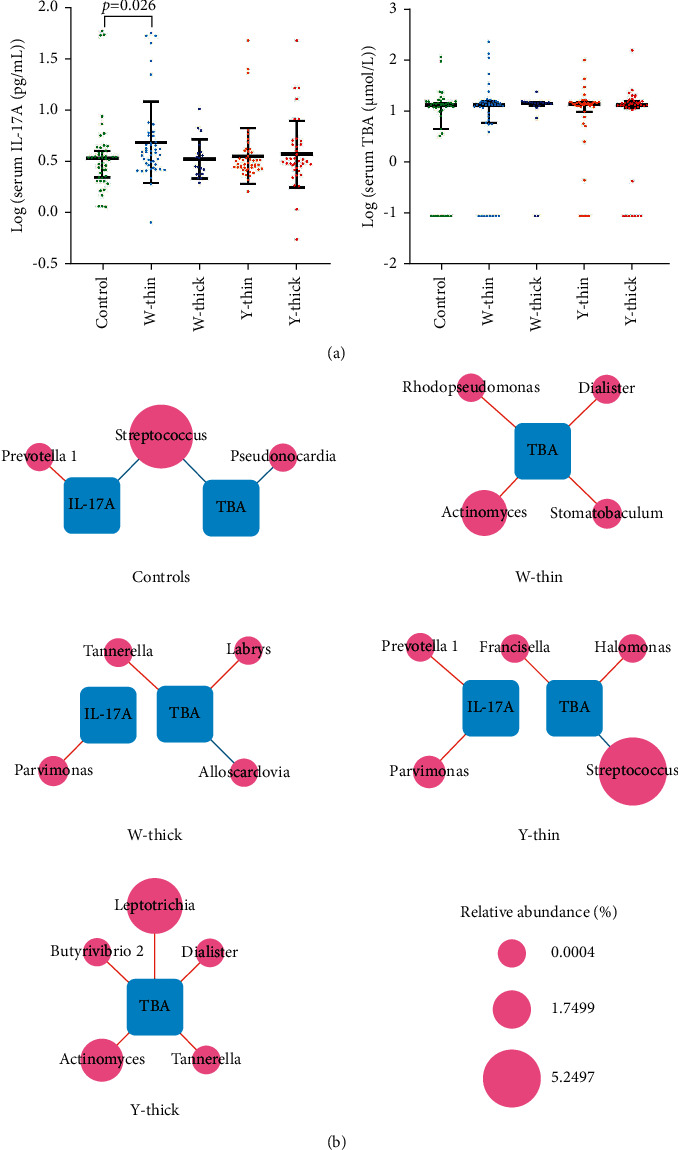
Correlation among serum IL-17A, TBA, and TCs distinct genera. (a) According to the Mann–Whitney *U*-test, the serum IL-17A was remarkably enhanced in the PLUGT cases with W-thin TC (*P* < 0.05); however, no discrepancies were detected in the serum TBA levels. (b) The commensal networks among serum IL-17A, TBA, and the distinct genera in the five groups. The size of node indicates the relative abundance, the red edges mean positive relationships, and the blue edges mean negative relationships (*P* < 0.05).

Serum levels of IL-17A and TBA were detected using ELISA. The Mann–Whitney *U* analysis was employed to ascertain if any two groups (PLUGT groups and controls) are considerably distinct from each other in terms of IL-17A and TBA levels. It was found that IL-17A levels in the W-thin group were substantially higher than those in the control group (*P* < 0.05), although there existed no remarkable discrepancy in serum TBA levels between the groups (*P* > 0.05; Figure 6(a)). Spearman's correlation analysis was employed for exploring the potential relationship between serum IL-17A, TBA, and the distinct genera (Table S2), and the meaningful relationships (*P* < 0.05) were created into symbiotic networks (Figure 6(b)). The results showed that *Parvimonas* positively associated with serum IL-17A in W-thick and Y-thin coatings. *Dialister* positively associated with serum TBA in W-thin and Y-thick coatings. Significantly, the oral dominant bacteria *Streptococcus* negatively correlated with both serum IL-17A and TBA in the control group, while negatively correlated with only serum TBA in Y-thin coating.

## 4. Discussion

Tongue diagnosis, or more accurately TC diagnosis, is one of traditional Chinese medicine's most essential diagnostic instruments. On the surface of the tongue, TC appears as a grayish-white deposit. The tongue coating is made up of blood cells, epithelial cells, vascular endothelial cells, bacteria, and a variety of metabolites.

Since the TC microbiota was firstly profiled by Jiang et al. [34], which indicated that different TCs link certain pathological status even in the same disease and occupy unique microbiota and commensal networks. Then, a great deal of literature presented the relationship between TC microbiota and systemic diseases, especially many digestive cancers and precancerous lesions [25]. As everyone knows, TC microorganisms can enter the digestive tract with saliva and colonize the mucosal epithelium of the digestive tract. Therefore, here assumes that TC microbiota correlates to PLUGT progression, and analyzed the characteristics of the bacterial community of four common TCs in PLUGT patients to provide new potential biomarkers for accurate PLUGT diagnosis.

This exploration revealed that the bacterial richness and diversity of PLUGT cases were substantially superior to those of the control group. The bacterial richness and diversity in the W-thin, Y-thick, and Y-thin groups in PLUGT patients were remarkably superior to those in the control group. However, this result is inconsistent with the results of the already published investigations [19, 22, 35]. For example, Xu et al. [19] discovered that there existed no substantial discrepancy in the alpha diversity of microbiota in common TCs in GC patients, Xu et al. [22] found that GC patients had greater bacterial richness and lower bacterial diversity than those in the control group, Hu et al. [35] divulged that GC cases with thick TC demonstrated lesser bacterial diversity than the patients with thin TC and healthy controls. These inconsistent results may be related to complex factors including sex, age, region, and dietary habits.

In this study, it was found that the microbial community structure of PLUGT patients was similar to that of GC patients in previous studies [19, 35]. This finding suggests that tongue-coating microorganisms have potential diagnostic value for PLUGT. At the phylum level, cluster analysis revealed that the W-thin and W-thick groups were clustered together and the Y-thin and Y-thick groups were clustered together in PLUGT patients. Cheng et al. [36] found that the difference between white and yellow coatings in early gastric cancer patients was greater than that between thin and thick coatings. Jiang et al. [34] found that there were significant differences in the microorganisms of white-greasy and yellow-dense coatings in chronic gastritis (CG) patients. The aforementioned results indicate that the tongue coating color changes were related to the microbial community structure of the tongue coating. The changes in the microbial community in this study were similar to those observed in the esophageal mucosa of esophageal mucosal metaplasia patients [37]. The results suggested that the microbial community of the upper digestive tract, and the tongue coating might have similar changes in the progression of upper gastrointestinal cancer.

TCM believes that white and yellow coating represents cold and hot syndrome, respectively. In this study, *Leptotrichia* was a potential biomarker of Y-thin and Y-thick groups in PLUGT cases, and the relative abundance of *Leptotrichia* was considerably greater in them than in the control group. *Leptotrichia* has extensive genetic diversity. To date, six species belonging to this genus have been validly established [38]. *Leptotrichia* is an opportunistic bacterium that causes opportunistic infections when immune function is compromised by tumor chemotherapy and granulocytopenia disease [38–40]. *Leptotrichia* has strong immunogenicity and can stimulate a mucosal immune response, and anti-*Leptotrichia* antibody can be detected in serum [38]. It is speculated that the immune-inflammatory response resulting from *Leptotrichia* may promote carcinogenesis of the upper digestive mucosa. For instance, the relative abundance of *Leptotrichia* was significantly increased in colon cancer tissue, GC tissue, and mouthwash of pancreatic cancer patients [41–43]. A study showed that *Streptococcus* and *Leptotrichia* in saliva were positively correlated with the risk of pancreatic adenocarcinoma (PDAC), whereas *Veillonella* and *Neisseria* in saliva were negatively correlated with the risk of PDAC [44]. Furthermore, nonparametric test analysis found that the relative abundance of *Campylobacter* in Y-thin and Y-thick groups was significantly increased. Chen and Xie [45] found that rheumatoid arthritis (RA) activity was positively correlated with the degree of yellow tongue coating. Wang et al. [46] believed that RA patients with damp-heat obstruction regularly exhibited high disease activity. Damp and heat are often the causes of yellow-greasy coating formation [47]. Therefore, the tongue-coating type is closely related to RA activity. Previous studies found that the abundance of *Actinomyces* and *Campylobacter* in RA patients was significantly higher than that in healthy controls [48, 49]. The aforementioned results suggest that *Leptotrichia* and *Campylobacter* could be promising biomarkers of yellow coating and are related to the hot syndrome.

TCM regards the image of the tongue as a mirror image of the body's visceral functions. Changes in tongue coating reflect the pathophysiological status of the body. Tongue coating can be used to diagnose diseases and symptoms as a unique diagnostic indicator [25]. In this study, LEfSe analysis was used to analyze the unique biomarkers of regular tongue coatings in PLUGT patients. The unique biomarkers of the W-thin group include *Psychrobacter*, *Pseudoramibacter*, and *Aeromonas*. Kwon et al. [50] discovered that the abundance of *Psychrobacter* diminished in cervical swabs from cervical cancer patients. Gao et al. [51] explored that the abundance of *Psychrobacter* was diminished in cancerous tissues from colorectal cancer patients. Jia et al. [52] revealed that compared with intrahepatic cholangiocarcinoma (ICC) patients with vascular invasion (VI), the abundance of *Pseudoramibacter* in the intestines of ICC patients without VI was significantly higher; *Pseudoramibacter* was negatively correlated with plasma tauroursodeoxycholic acid, which was positively associated with tumor number and negatively associated with survival time. *Aeromonas* is one of the commonly occurring gastrointestinal pathogens associated with infections in animals and humans [53]. Moreover, numerous extracellular proteins, comprising hemolysin, endotoxins, and adhesion factors, are involved in the pathogenesis of *Aeromonas* [54–56]. Gao et al. [57] found that sleep deprivation significantly increased intestinal *Aeromonas* in mice. The unique biomarkers of the W-thick group include *C. sensu stricto 1, Kingella*, and *Thermus*. The relative abundance of intestinal *C. sensu stricto 1*increased in obese patients and was significantly positively correlated with body weight, blood lipid, and uric acid (*P* < 0.0 5) [58]. The increased oral abundance of Kingella is linked to a lower risk of head and neck squamous cell cancer (HNSCC), which could have implications for cancer prevention [59]. The genus *Thermus* is more abundant in tissues from developed lung cancer (IIIB, IV) patients, and it can perform a task in tumor development by means of different microbial functions, such as aldosterone-regulated sodium reabsorption, decreased signal transduction, amino acid metabolism, increased excretory system, and amoebiasis pathways [60].

Using KEGG predictive function analysis to explore the molecular mechanism of common TC formation in PLUGT patients, we found that the RLR signaling pathway in the W-thin group and cell motility and secretion in the Y-thick group were significantly enhanced. RLRs are important initiators of the innate immune response to RNA virus infection [61]. The RLR activates downstream transcription factors, leading to the generation of type-1 interferon and expression of an antiviral gene, eliciting an intracellular immune response capable of controlling viral infection [62]. Zhao et al. [63] found that cell motility (bacterial motility proteins) and membrane transport (secretion system) were enriched in chronic hepatitis B cases with yellow tongue coatings. The results provide clues for further exploration of the biological mechanism of the tongue coating that reflects the body's pathophysiological state and indicate that yellow coating could be substantially susceptible to bacterial colonization.

IL-17A performs a key task in host defense against fungal and bacterial infections, as well as in the development of autoimmunity, inflammation, and tumors. IL-17A greatly enhances protective immune responses in epithelial cells and keratinocytes by inducing the production of G-CSF, CXC chemokines, and antimicrobial peptides [64]. Our last research found that GC patients had an enhanced level of serum IL-17A [32], and here found that the PLUGT patients with W-thin TC had a greater serum IL-17A level than the controls (*P* < 0.05). The commensal networks presented that serum IL-17A positively correlated to *Parvimonas* in the W-thick group and Y-thin group and positively correlated to *Prevotella 1* in Y-thin group. Oral bacterium *Parvimonas* was found to be overexpressed in the gastric mucosa of GC patients and to have high centrality in the GC ecological network, indicating that it might act as a backbone for niche-specific connections and may exert considerable impact on the GC microbial ecology [65]. In addition, Zhao et al. [63] found an enhanced abundance of *Prevotella* in esophagitis or Barrett's oesophagus. Iwakura et al. [64] found salivary *Parvimonas micra* and *Prevotella* sp. were increased in periodontitis patients. A cohort study found that advanced periodontitis was associated with elevated risks of EC [65], and many kinds of research [66, 67] proved that a high level of oral IL-17 promoted the development of periodontitis via its pro-inflammatory and osteoclastogenic properties [68]. These results could assume that W-thick and Y-thin coatings may represent the oral pathogenic trait and inflammation, and simultaneously reflect the progress of upper digestive tract cancer.

Bile acids have a vital role in the development of gastric intestinal metaplasia (IM) and carcinogenesis of the gastric mucosa. Multiple studies have shown that cases with esophageal adenocarcinomas demonstrated lesser levels of IL-17A than healthy controls [29–31]. As our earlier study revealed that GC patients had substantially greater serum IL-17A levels than controls, while those with white coatings had remarkably lesser serum IL-17A levels than those with yellow coatings [32]. The current exploration discovered the association between unique TC bacteria and serum IL-17A and TBA levels, as well as the microbiological characteristics and potential biomarkers of common TC (W-thin, W-thick, Y-thin, and Y-thick) in PLUGT patients. According to the obtained results, the promoted bacteria were found to be synergistically proliferative in the TCs of PLUGT patients. In addition, the different TCs exhibited distinct commensal networks, whereas the common TCs were associated with serum IL-17A and TBA via specific bacteria. The findings are valuable because they can potentially furnish useful solutions to achieve a speedy and noninvasive diagnosis of PLUGT, as well as exemplify the scientific meaning of tongue diagnosis, thereby assisting in the differentiation and treatment of TCM syndromes.

## 5. Conclusion

This study examined the microbiological properties of four common TCs in PLUGT patients, as well as the relationship between the different TC bacteria and blood IL-17A and TBA, The potential biomarkers of common TC (W-thin, W-thick, Y-thin, and Y-thick) in PLUGT patients were also investigated. The findings add valuable information to our understanding of the biological mechanism of tongue coating development. Nevertheless, validation in a large population is needed due to the heterogeneity in individual habits, which impacts the universality of the study results. The results, however, are significant because they may provide effective ways for obtaining a quick and noninvasive diagnosis of PLUGT. Furthermore, the findings suggested that TC differentiation could provide a more accurate diagnostic for PLUGT clinical administration.

## Data Availability

Data will be provided on request to the corresponding author.

## Supplementary Materials

Supplementary Materials
*Figure S1*. The significantly different predictive functions of TC microbiota between PLUGT patients and controls. *Figure S2*. LDA analysis of predictive functions of TC microbiota in PLUGT patients based on controls: (A) W-thin group, (B) Y-thin group, (C) Y-thick group, and (D) Venn analysis among the common TCs in PLUGT patients. *Table S1*. The alpha diversity of tongue-coating microbiota in the controls and common tongue coatings in PLUGT patients (relative abundance, median (P25, P75) %). *Table S2.* Mann–Whitney *U*-test of the distinct genera between PLUGT patients and the controls.
