# Supplementary material for: Microbial Characteristics of Common Tongue Coatings in Patients with Precancerous Lesions of the Upper Gastrointestinal Tract
Source: J Healthc Eng. 2022 Apr 18;2022:7598427. doi: 10.1155/2022/7598427 (PMC9038387; doi:10.1155/2022/7598427)
Supplement: Supplementary Materials — Figure S1. The significantly different predictive functions of TC microbiota between PLUGT patients and controls. Figure S2. LDA analysis of predictive functions of TC microbiota in PLUGT patients based on controls: (A) W-thin group, (B) Y-thin group, (C) Y-thick group, and (D) Venn analysis among the common TCs in PLUGT patients. Table S1. The alpha diversity of tongue-coating microbiota in the controls and common tongue coatings in PLUGT patients (relative abundance, median (P25, P75) %). Table S2. Mann–Whitney U-test of the distinct genera between PLUGT patients and the controls. [file 7598427.f1.zip › 7598427.f1/Table S2.pdf]

Table S2. Mann–Whitney *U* test of the distinct genera between PLUGT patients and the control group

							Relative abunda
genus	Controls (n=47)			Wthin (n=47)			W
	50%	25%	75%	50%	25%	75%	50%
Variovorax	0.000	0.000	0.000	0.000	0.000	0.000	0.000
Taonella	0.000	0.000	0.000	0.000	0.000	0.000	0.000
Tannerella	0.018	0.009	0.045	0.027	0.009	0.072	0.054
Streptococcus	5.294	3.499	7.972	7.458	4.392	10.632	4.392
Stomatobaculum	0.108	0.054	0.207	0.198	0.126	0.298	0.153
Sphingomonas	0.000	0.000	0.000	0.000	0.000	0.000	0.000
Simonsiella	0.000	0.000	0.000	0.000	0.000	0.000	0.000
Selenomonas 3	0.081	0.027	0.207	0.171	0.045	0.334	0.162
Ruminococcaceae UCG-014	0.117	0.036	0.216	0.144	0.054	0.352	0.207
Rhodopseudomonas	0.000	0.000	0.000	0.000	0.000	0.009	0.000
Ralstonia	0.000	0.000	0.000	0.000	0.000	0.027	0.000
Pseudonocardia	0.000	0.000	0.000	0.000	0.000	0.000	0.000
Pseudomonas	0.000	0.000	0.000	0.000	0.000	0.000	0.000
Prevotella 6	0.361	0.072	0.956	0.911	0.216	1.560	0.550
Prevotella 1	0.000	0.000	0.009	0.000	0.000	0.009	0.000
Parvimonas	0.469	0.099	1.001	0.180	0.027	0.460	0.225
Olsenella	0.000	0.000	0.000	0.000	0.000	0.000	0.000
Nocardia	0.000	0.000	0.000	0.000	0.000	0.000	0.000
Nesterenkonia	0.000	0.000	0.000	0.000	0.000	0.000	0.000
Methylobacterium	0.000	0.000	0.000	0.000	0.000	0.000	0.000
Mesorhizobium	0.000	0.000	0.000	0.000	0.000	0.018	0.000
Leptotrichia	1.028	0.307	2.660	2.651	1.091	6.998	1.704
Lachnoanaerobaculum	0.343	0.234	0.487	0.550	0.307	0.758	0.478
Labrys	0.000	0.000	0.009	0.000	0.000	0.009	0.000
Kingella	0.000	0.000	0.009	0.000	0.000	0.009	0.009
Halomonas	0.000	0.000	0.000	0.000	0.000	0.000	0.000
Francisella	0.000	0.000	0.000	0.000	0.000	0.000	0.000
Dialister	0.009	0.000	0.018	0.009	0.000	0.027	0.009
Defluviitaleaceae UCG-011	0.009	0.000	0.036	0.009	0.000	0.018	0.009
Clostridium sensu stricto 1	0.000	0.000	0.000	0.000	0.000	0.000	0.000
Christensenellaceae R7 group	0.000	0.000	0.000	0.000	0.000	0.000	0.000
Cardiobacterium	0.000	0.000	0.009	0.000	0.000	0.009	0.009
Campylobacter	0.568	0.225	0.857	0.685	0.415	1.037	0.767
Butyrivibrio 2	0.009	0.000	0.018	0.009	0.009	0.027	0.009
Bradyrhizobium	0.000	0.000	0.000	0.000	0.000	0.009	0.000
Alloscardovia	0.000	0.000	0.000	0.000	0.000	0.000	0.000
Actinomyces	1.641	0.180	3.210	3.219	0.803	4.527	2.723
Acidovorax	0.000	0.000	0.000	0.000	0.000	0.000	0.000
[Eubacterium] yurii group	0.090	0.018	0.162	0.036	0.009	0.090	0.063

Comparison with the controls, a refers to W-thin, b refers to W-thick, c refers to Y-thin, d refers to Y-thick.

.

ınce, median (P25, P75) %

/thick (n=19)		Ythin (n=47)			Ythick (n=40)			Z^a	P^a
25%	75%	50%	25%	75%	50%	25%	75%		
0.000	0.000	0.000	0.000	0.000	0.000	0.000	0.000	2.381	0.017
0.000	0.000	0.000	0.000	0.000	0.000	0.000	0.000	2.033	0.042
0.000	0.099	0.036	0.009	0.081	0.045	0.018	0.072	1.306	0.192
3.156	6.403	5.582	3.986	9.748	5.956	3.724	8.641	2.284	0.022
0.045	0.451	0.126	0.054	0.451	0.234	0.129	0.564	2.720	0.007
0.000	0.000	0.000	0.000	0.000	0.000	0.000	0.000	1.718	0.086
0.000	0.009	0.000	0.000	0.000	0.000	0.000	0.000	2.408	0.016
0.018	0.496	0.126	0.036	0.243	0.176	0.063	0.300	1.737	0.082
0.072	0.406	0.126	0.036	0.234	0.158	0.056	0.304	1.328	0.184
0.000	0.000	0.000	0.000	0.000	0.000	0.000	0.000	3.680	0.000
0.000	0.009	0.000	0.000	0.027	0.000	0.000	0.009	1.326	0.185
0.000	0.009	0.000	0.000	0.009	0.000	0.000	0.009	2.394	0.017
0.000	0.000	0.000	0.000	0.000	0.000	0.000	0.000	2.157	0.031
0.198	1.290	0.523	0.171	1.425	0.649	0.097	1.172	2.280	0.023
0.000	0.009	0.000	0.000	0.009	0.000	0.000	0.009	1.214	0.225
0.090	0.577	0.460	0.036	0.938	0.428	0.151	1.407	2.088	0.037
0.000	0.000	0.000	0.000	0.000	0.000	0.000	0.000	1.422	0.155
0.000	0.000	0.000	0.000	0.000	0.000	0.000	0.000	2.517	0.012
0.000	0.000	0.000	0.000	0.000	0.000	0.000	0.000	0.000	1.000
0.000	0.000	0.000	0.000	0.009	0.000	0.000	0.000	2.124	0.034
0.000	0.018	0.000	0.000	0.009	0.000	0.000	0.007	1.847	0.065
0.685	6.628	1.957	0.541	5.762	2.390	0.760	7.401	2.870	0.004
0.271	0.694	0.505	0.316	0.721	0.622	0.327	0.845	2.818	0.005
0.000	0.000	0.000	0.000	0.018	0.000	0.000	0.009	1.452	0.146
0.000	0.018	0.000	0.000	0.009	0.000	0.000	0.009	0.383	0.702
0.000	0.000	0.000	0.000	0.000	0.000	0.000	0.000	1.351	0.177
0.000	0.000	0.000	0.000	0.000	0.000	0.000	0.000	1.422	0.155
0.000	0.054	0.009	0.000	0.036	0.027	0.009	0.061	0.534	0.593
0.000	0.054	0.009	0.000	0.027	0.009	0.000	0.027	1.982	0.047
0.000	0.000	0.000	0.000	0.000	0.000	0.000	0.000	0.000	1.000
0.000	0.000	0.000	0.000	0.000	0.000	0.000	0.000	2.285	0.022
0.000	0.009	0.000	0.000	0.009	0.000	0.000	0.009	0.542	0.587
0.442	1.208	0.803	0.433	1.263	0.933	0.431	1.364	1.823	0.068
0.000	0.054	0.009	0.000	0.027	0.018	0.000	0.043	2.205	0.027
0.000	0.000	0.000	0.000	0.009	0.000	0.000	0.007	2.406	0.016
0.000	0.000	0.000	0.000	0.000	0.000	0.000	0.000	0.803	0.422
0.397	4.825	2.426	0.658	4.752	2.047	0.487	4.103	2.480	0.013
0.000	0.000	0.000	0.000	0.000	0.000	0.000	0.000	2.460	0.014
0.000	0.135	0.063	0.009	0.171	0.063	0.009	0.133	2.395	0.017

Z^b	P^b	Z^c	P^c	Z^d	P^d
1.497	0.134	2.375	0.018	1.894	0.058
0.000	1.000	1.751	0.080	1.084	0.278
1.782	0.075	1.702	0.089	2.692	0.007
0.630	0.529	0.904	0.366	0.686	0.493
1.304	0.192	1.045	0.296	3.140	0.002
0.906	0.365	2.196	0.028	1.047	0.295
0.909	0.364	0.531	0.595	0.974	0.330
1.305	0.192	1.627	0.104	2.340	0.019
1.998	0.046	0.121	0.904	1.517	0.129
2.767	0.006	2.939	0.003	2.206	0.027
0.526	0.599	2.510	0.012	1.301	0.193
2.720	0.007	2.983	0.003	2.699	0.007
0.636	0.525	0.584	0.560	1.150	0.250
1.494	0.135	1.237	0.216	1.516	0.129
0.111	0.912	2.283	0.022	1.680	0.093
1.368	0.171	0.322	0.748	0.635	0.526
2.242	0.025	1.000	0.317	1.542	0.123
1.573	0.116	2.033	0.042	1.084	0.278
1.573	0.116	2.285	0.022	1.542	0.123
0.540	0.589	2.524	0.012	1.692	0.091
0.952	0.341	2.326	0.020	0.809	0.418
1.834	0.067	2.280	0.023	2.283	0.022
1.948	0.051	2.833	0.005	3.395	0.001
0.481	0.631	2.264	0.024	1.403	0.160
1.974	0.048	0.776	0.438	0.359	0.720
2.038	0.042	0.559	0.576	1.552	0.121
1.573	0.116	2.033	0.042	0.000	1.000
0.096	0.923	0.433	0.665	2.710	0.007
0.174	0.862	1.166	0.243	0.585	0.558
2.242	0.025	0.000	1.000	1.084	0.278
0.625	0.532	0.727	0.467	0.476	0.634
2.041	0.041	0.525	0.600	0.169	0.866
1.537	0.124	2.443	0.015	2.572	0.010
0.369	0.712	0.745	0.456	1.733	0.083
1.312	0.190	2.771	0.006	2.037	0.042
0.284	0.776	2.033	0.042	0.272	0.786
1.657	0.098	2.106	0.035	1.393	0.164
0.668	0.504	0.584	0.559	2.195	0.028
1.250	0.211	0.762	0.446	1.397	0.163
