# Supplementary figures and images for: Microbial Characteristics of Common Tongue Coatings in Patients with Precancerous Lesions of the Upper Gastrointestinal Tract
Source: J Healthc Eng. 2022 Apr 18;2022:7598427. doi: 10.1155/2022/7598427 (PMC9038387; doi:10.1155/2022/7598427)

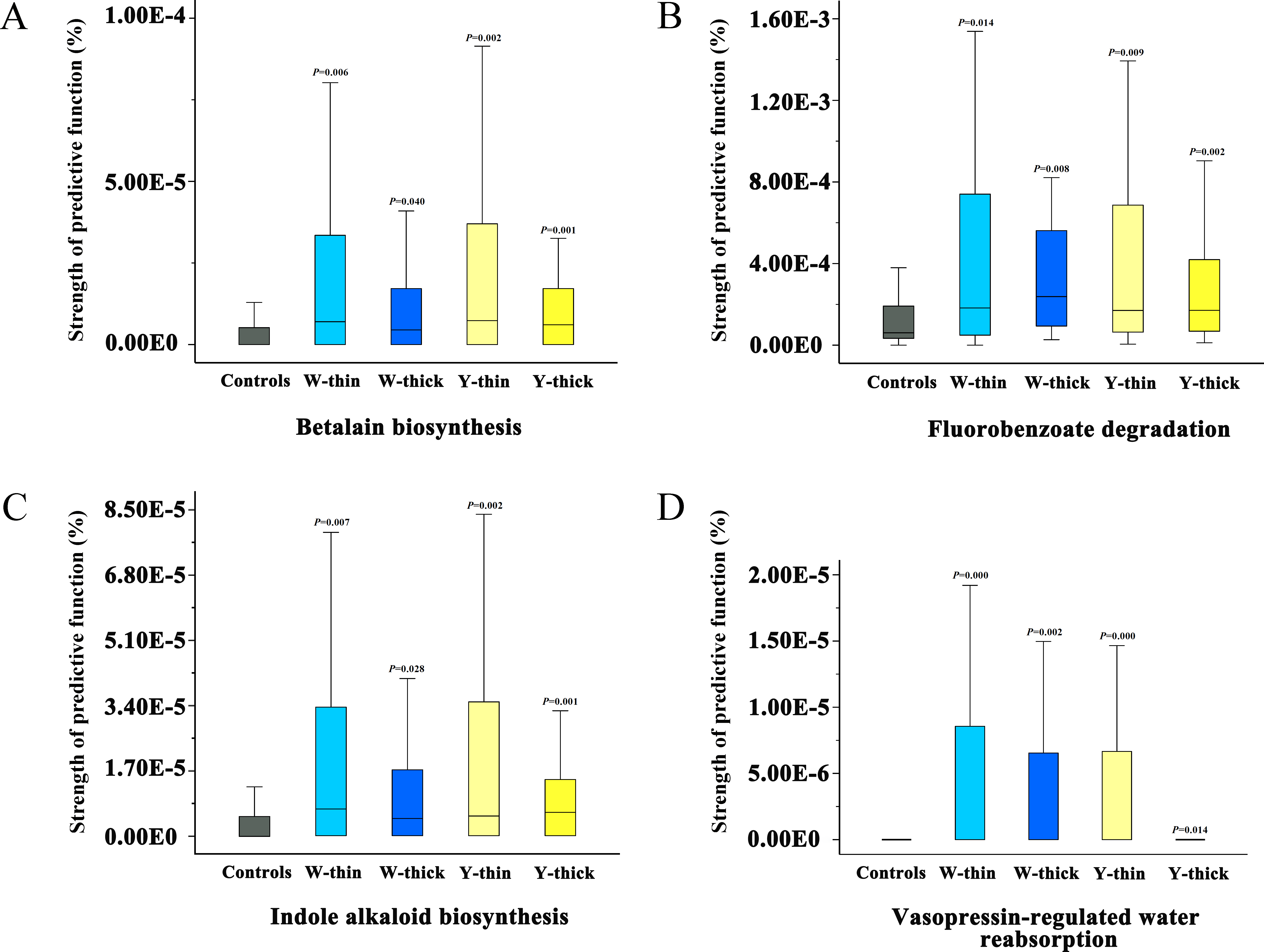

Supplement: Supplementary Materials — Figure S1. The significantly different predictive functions of TC microbiota between PLUGT patients and controls. Figure S2. LDA analysis of predictive functions of TC microbiota in PLUGT patients based on controls: (A) W-thin group, (B) Y-thin group, (C) Y-thick group, and (D) Venn analysis among the common TCs in PLUGT patients. Table S1. The alpha diversity of tongue-coating microbiota in the controls and common tongue coatings in PLUGT patients (relative abundance, median (P25, P75) %). Table S2. Mann–Whitney U-test of the distinct genera between PLUGT patients and the controls. [file 7598427.f1.zip › 7598427.f1/Figure-S1.png]

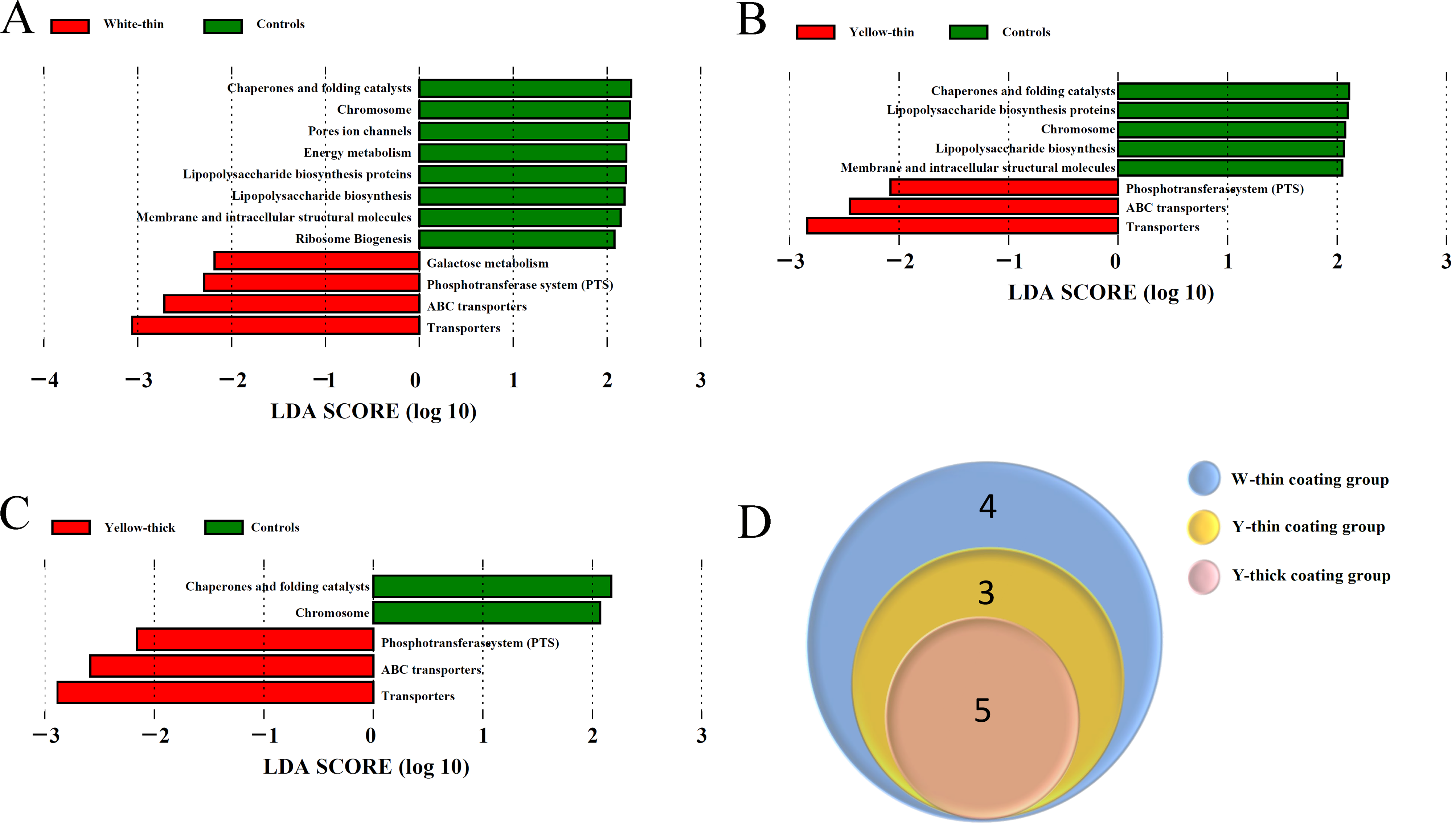

Supplement: Supplementary Materials — Figure S1. The significantly different predictive functions of TC microbiota between PLUGT patients and controls. Figure S2. LDA analysis of predictive functions of TC microbiota in PLUGT patients based on controls: (A) W-thin group, (B) Y-thin group, (C) Y-thick group, and (D) Venn analysis among the common TCs in PLUGT patients. Table S1. The alpha diversity of tongue-coating microbiota in the controls and common tongue coatings in PLUGT patients (relative abundance, median (P25, P75) %). Table S2. Mann–Whitney U-test of the distinct genera between PLUGT patients and the controls. [file 7598427.f1.zip › 7598427.f1/Figure-S2.png]
